# Handheld Weights as an Effective and Comfortable Way To Increase Exercise Intensity of Physical Activity in Virtual Reality: Empirical Study

**DOI:** 10.2196/39932

**Published:** 2022-11-23

**Authors:** Jacek Polechoński, Anna Zwierzchowska, Łukasz Makioła, Dorota Groffik, Karolina Kostorz

**Affiliations:** 1 Institute of Sport Sciences The Jerzy Kukuczka Academy of Physical Education in Katowice Katowice Poland; 2 Student Scientific Circle of Physical Activity and Tourism in Virtual Reality “ACTIVE VR” The Jerzy Kukuczka Academy of Physical Education in Katowice Katowice Poland

**Keywords:** immersive virtual reality, virtual reality, health-related physical activity, intensity of physical activity, active video games, serious games

## Abstract

**Background:**

In recent years, there has been a growing interest in active virtual reality games (AVRGs) that provide entertainment and encourage more physical activity (PA). Since playing AVRGs involves primarily arm movements, the intensity of this form of PA may not be sufficient for health benefits. Therefore, it is worth looking for virtual entertainment solutions that are comfortable for users and at the same time increase physical exercise.

**Objective:**

The main objective of this study was to evaluate the effect of external loading of the arms in the form of handheld weights (HHWs) on exercise intensity in users playing a popular AVRG. The results obtained in the study were compared with the PA recommendations for health. The study also assessed the perceptions of the users about the attractiveness and usefulness of this type of exercise and discomfort caused by additional load on the arms.

**Methods:**

The study covered 17 young adults aged 18 to 25 years playing an AVRG (Beat Saber) with no arm load and with HHWs (0.5 kg). A PlayStation 4 PRO console (Sony) with accessories including a head-mounted display and controllers was used in the study. PA intensity was evaluated using a heart rate monitor based on the percentage of maximal heart rate (% HR_max_). The usability, attractiveness, and comfort perceived during exercise by users were evaluated using a survey questionnaire.

**Results:**

The measurements showed that the mean % HR_max_ in participants playing Beat Saber without HHWs was significantly lower (*P*<.001; Cohen *d*=1.07) than that observed when playing with HHWs. It should be emphasized that with no additional load, the intensity of PA was low (mean 63.7% HR_max_, SD 9.3% HR_max_), while with the upper limb load, it increased to a moderate level (mean 67.1% HR_max_, SD 10.3% HR_max_), which is recommended for health benefits. The survey conducted in the study showed that HHWs (0.5 kg) attached to the wrists did not disturb Beat Saber players.

**Conclusions:**

Since PA in most of the modern AVRGs primarily involves upper limb movements, the use of HHW seems to be a simple and effective way to increase exercise intensity, especially because, as reported by the study participants, such a procedure does not cause discomfort while using the application.

## Introduction

The last decade has seen unprecedented advances in immersive virtual reality (VR) [1]. There is no doubt that technology has revolutionized the way the user interacts with the digital world. Nowadays, with modern head-mounted displays (HMDs) and custom software, this state-of-the-art technology simulates a real-world environment by providing the user with 3D experience without discomfort [2]. Increasing computing power, improved graphics, greater processing speed, and better internet connectivity have resulted in better access and higher consumer demand for VR [3]. Consequently, VR technology is increasingly being used in various areas of life and science, including health care, rehabilitation, psychology, education, engineering, manufacturing, and entertainment [2,4-10]. The development of information technology and the ever-increasing popularity of video games are often associated with the reduction of a person's physical activity (PA) in favor of prolonged sitting in front of a computer, resulting in poor well-being and progressive deterioration in the physical and mental health status [11-14] Therefore, in recent years, there has been a growing interest in active virtual reality games (AVRGs), which provide entertainment and encourage PA [15-25]. AVRGs are video games that require users to perform physical exercise [26].

Not surprisingly, there has been a steady increase in the number of scientific studies that verify, compare, and discuss interventions using VR. With the development of modern civilization and sedentary lifestyles, the use of VR in sports and in promoting PA is particularly important [15,27-30]. However, the level of PA intensity is extremely important to achieving health benefits. According to the guidelines of the World Health Organization (WHO), PA intensity should be at least moderate [31]. However, published research findings are inconclusive. Some researchers point to moderate to high levels of exercise intensity in those playing AVRGs, especially when using exercise machines (eg, cycle ergometers, treadmill) [16,18,19,22,23]. Locomotion has been shown to promote exercise intensity and enhance the immersion experience. Consequently, research has been undertaken to analyze physical exercise in VR [32-35]. It was found that exercise intensity using exercise machines in VR can be higher compared to similar PA during conventional training sessions [20].

There are also reports that PA in VR is characterized by rather low intensity, especially when the game is played using room-scale tracking, in which the user moves in a limited space, and movements are mainly performed with the upper limbs [36]. Unfortunately, this is the most common way to use HMDs. The question arises: What can be done to increase PA intensity when using movement applications in VR without having to use expensive and bulky indoor trainers? Given that standard controllers are operated primarily by arm movements, it was assumed that a simple solution might be to add a small additional load on the upper limbs using Velcro-fastened handheld weights (HHWs) on the wrists. This concept inspired the experiment described in this paper. It should be added that sets of weights fastened with Velcro are popular equipment used in various forms of PA, such as aerobics, jogging, general fitness exercises, and rehabilitation [37-39]. However, to date, HHW has not been used in studies on physical exercise intensity in VR.

Considering all of the aforementioned issues, the main aim of this study is to evaluate the effect of external arm loading on the intensity of PA in young adults playing a popular AVRG (Beat Saber). It was assumed that the use of HHW would facilitate the achievement of PA intensity at the level recommended for health benefits. Another assumption was that the load applied would not reduce the player's perception of comfort during the game.

## Methods

### Participants

The study was conducted on a group of 17 participants aged 18 to 25 years: 7 women (mean age 20.7 years, SD 2.6 years; mean body height 169.9 cm, SD 6.3 cm; mean body weight 62.4 kg, SD 7.7 kg) and 10 men (mean age 19.6 years, SD 2.0 years; mean body height 174.7 cm, SD 7.8 cm; mean body weight 60.2 kg, SD 7.7 kg). They were recruited from participants of the Silesian Festival of Science held at the International Convention Center in Katowice, Poland, where hardware and software that allow players to perform PA in VR were presented. We selected a group of young adults who volunteered to participate in the study, were able to use VR technology, and did not require additional training in the use of VR equipment. It should also be noted that people of this age are the most frequent users of VR headsets. All participants signed a consent to participate in the experiment and were informed about the purpose and procedures of the study. Furthermore, the following inclusion criteria were used: good general health status, no medical contraindications to participation in the study (specifically, no epileptic history or motion sickness), no physical limitations (eg, injuries) that might restrict PA in VR, and not taking any agents that affect heart rate.

### Ethics Approval

The study was conducted according to the guidelines of the Declaration of Helsinki and reviewed and approved by the Research Ethics Committee of the Jerzy Kukuczka Academy of Physical Education in Katowice (protocol #9/2018). All participants took part in the study voluntarily and could discontinue their participation at any time.

### Research Tools and Procedures

**Figure 1:**
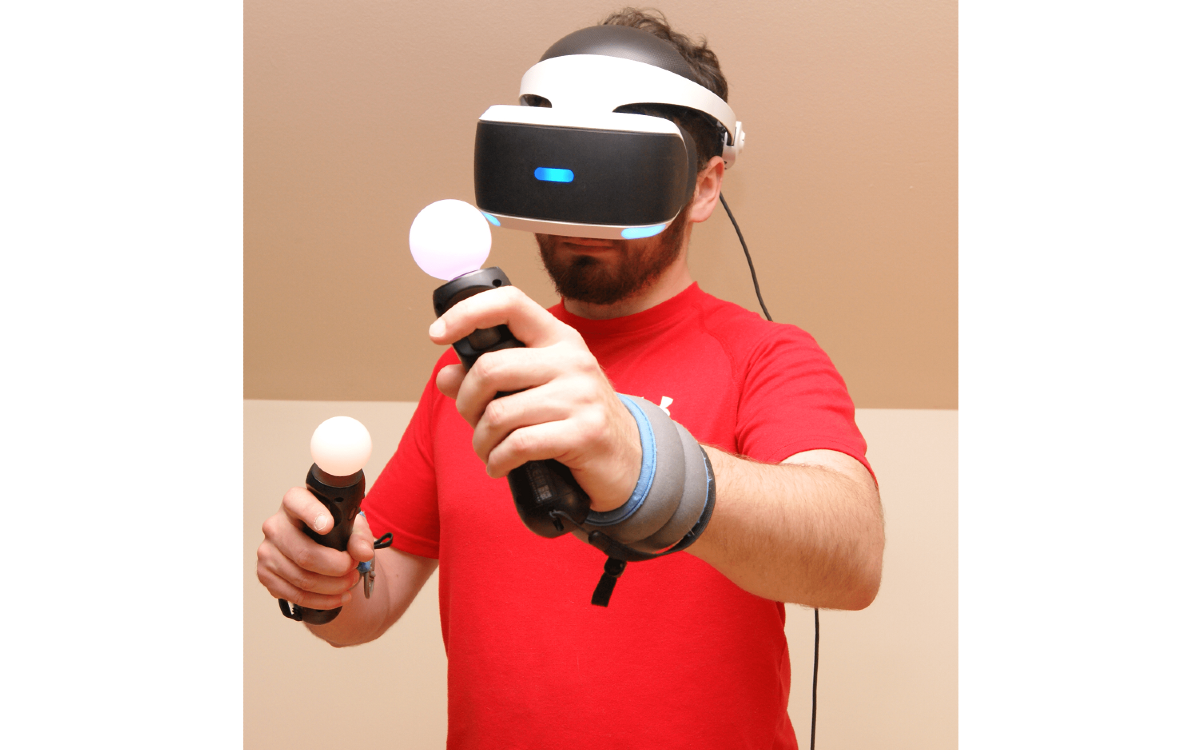
Participant playing an active video game (Beat Saber) in immersive virtual reality with handheld weights.

**Figure 2:**
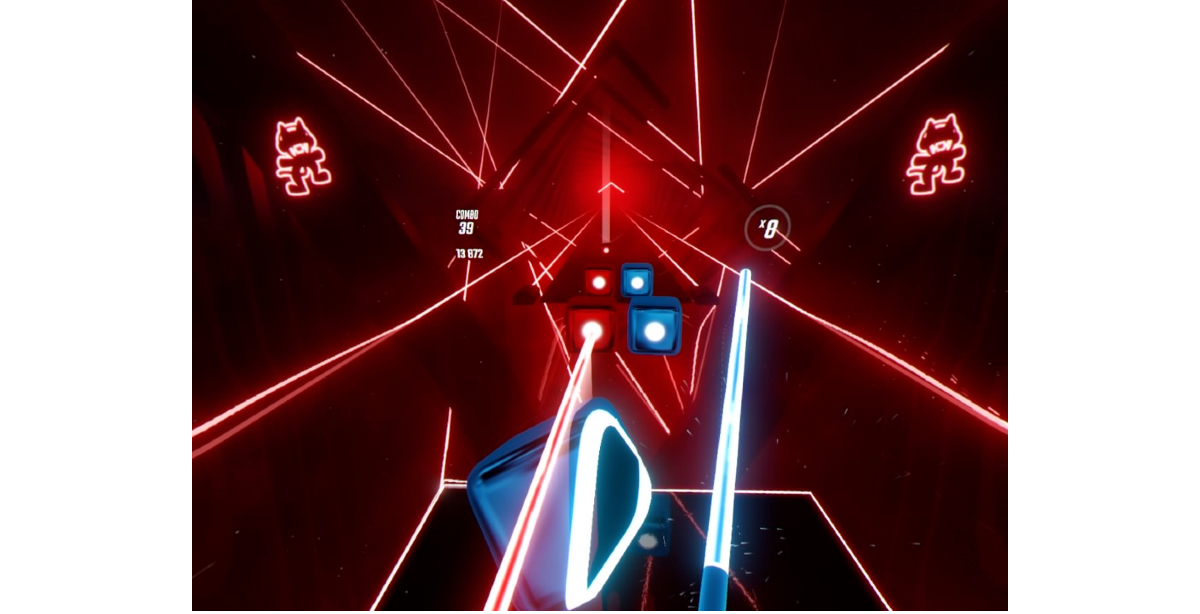
Gameplay of the Beat Saber game: print screen.

**Figure 3:**
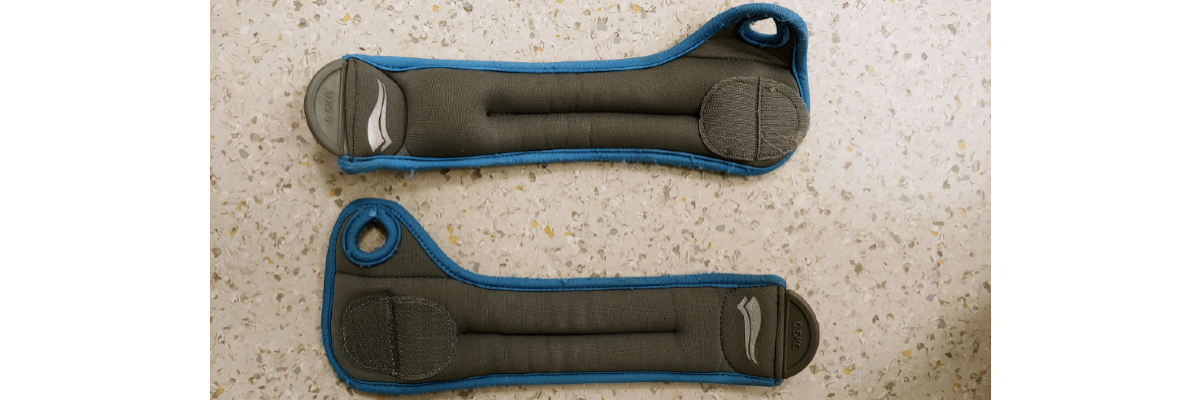
Handheld weights (0.5 kg) used in the study.

The PlayStation 4 PRO console (Sony) with accessories including an HMD and controllers was used in the study. The HMD, worn on the participant’s head, was used for displaying VR, while the controllers held in the hands allowed the participant to control the game. Figure 1 presents the user using the application. PA was evaluated during playing of the Beat Saber game, in which the player cuts through virtual cubes coming toward him or her with lightsabers to the beat of the music. The cutting direction is indicated by the arrows displayed on the objects. The moving cubes come in 2 colors, as do the virtual lightsabers held by the user. The participants played at the same difficulty level (normal) with an additional no-fail option to facilitate the procedure and ensure game continuity even after the number of mistakes allowed in the basic configuration had been exceeded. Furthermore, the no-arrow option was used for convenience to remove arrows from blocks and allow objects to be cut in any direction (Figure 2). The music track “Crab rave” was used and played in a loop to suit a 10-minute training session. Prior to the game, participants were trained on how to use the equipment and the application. The research procedure consisted of two 10-minute sessions of playing active video games. Each player participated in alternating sessions with and without arm loading. The order of sessions was counterbalanced. Every second person started the session with weights. There was a 30-minute rest between sessions. A 0.5-kg HHW fastened at the wrist of the right and left upper limbs was used to load the arms (Figure 3).

Heart rates were monitored during the playing of AVRGs using a Vantage V heart rate monitor (Polar Electro Oy). Exercise intensity was evaluated based on the average percentage of maximum heart rate (% HR_max_) obtained by each participant during the test. Before the experiment, the participant's HR_max_ was calculated from the following formula: 208 – 0.7 × age in years [40]. Exercise load was estimated based on the exercise intensity classification proposed by the American College of Sports Medicine [41]. According to the classification, average HR (HR_avg_) <64% HR_max_ is low intensity, 64% HR_max_ ≤ HR_avg_< 77% HR_max_ is moderate intensity, and HR_avg_ ≥ 77% HR_max_ is high intensity. The data obtained using this classification were compared to the criteria of health recommendations for the intensity of aerobic exercise, according to which exercise of at least moderate intensity is considered beneficial for health (≥64% HR_max_) [31]. Exercise intensity was also categorized into HR zones using the Polar Flow training analysis tool. The absolute time (in seconds) of HR spent in each of the following six zones was estimated as follows: I, <50% HR_max_; II, 50%-59% HR_max_; III, 60%-69% HR_max_; IV, 70%-79% HR_max_; V, 80%-89% HR_max_; and VI, ≥90% HR_max_. Calculations were performed for both training sessions to compare exercise intensity. The perceived exertion was estimated using the Borg Rating of Perceived Exertion (RPE) 6-20 scale [42], and its correlation with objective measurements was assessed.

After the completion of both games, our survey questionnaire consisting of 8 items (Table 1) was administered to gain information about the participants’ subjective perceptions of the attractiveness and usefulness of PA in VR and their comfort of exercising with HHWs. A 7-point Likert scale was used in the evaluation of the questionnaire. The respondents could choose between the following answers: “Strongly disagree,“ ”Disagree,“ ”Somewhat disagree,“ ”Neither agree nor disagree,“ ”Somewhat agree,“ ”Agree,“ and “Strongly agree.”

**Table 1:** Users' opinions on the attractiveness and usability of the Beat Saber game and the comfort of playing with HHWs (N=17).

Question	Strongly disagree, n (%)	Disagree, n (%)	Somewhat disagree, n (%)	Neither agree nor disagree, n (%)	Somewhat agree, n (%)	Agree, n (%)	Strongly agree, n (%)
1. Was playing VR^a^ attractive and enjoyable for you?	0 (0)	0 (0)	0 (0)	0 (0)	0 (0)	8 (47)	9 (53)
2. With the right hardware and software, would you regularly practice PA^b^ in VR?	1 (6)	0 (0)	0 (0)	0 (0)	1 (6)	8 (47)	7 (41)
3. Would you recommend practicing PA in VR to others?	0 (0)	0 (0)	0 (0)	0 (0)	2 (12)	11 (65)	4 (23)
4. Do you think that practicing PA in VR is more enjoyable than performing conventional training exercises?	0 (0)	1 (6)	1 (6)	5 (29)	0 (0)	5 (29)	5 (29)
5. Do you think practicing PA in VR can complement a person's leisure-time PA?	0 (0)	0 (0)	1 (6)	1 (6)	4 (23)	5 (30)	6 (35)
6. Do you think practicing PA in VR can satisfy a person's needs for leisure-time PA?”	0 (0)	0 (0)	2 (12)	2 (12)	3 (18)	8 (47)	2 (12)
7. Do you think practicing PA in VR can replace typical real-world forms of leisure-time PA?	1 (6)	1 (6)	5 (29)	2 (12)	7 (41)	1 (6)	0 (0)
8. Did you experience any discomfort while playing with HHW^c^?	12 (71)	2 (12)	2 (12)	0 (0)	1 (6)	0 (0)	0 (0)

^a^VR: virtual reality.

^b^PA: physical activity.

^c^HHW: handheld weight.

### Statistical Analysis

Statistical calculations were performed using Statistica v. 13 software (TIBCO Software Inc). Measurement data were analyzed using basic descriptive statistics. The results of the survey are presented as percentages. Arithmetic means, SDs, and differences in means were calculated. The data were tested for normal distribution using the Shapiro-Wilk test, whereas the significance of differences was evaluated using the *t* test or Wilcoxon test. Cohen *d* effect size was also calculated with <0.2=trivial effect, 0.2-0.5=small effect, 0.5-0.8=moderate effect, and >0.8=large effect [43]. Pearson correlation analysis was used to assess relationships.

## Results

### PA Intensity During the Playing of an AVRG (Beat Saber) With and Without HHWs in Light of Health Recommendations

**Figure 4:**
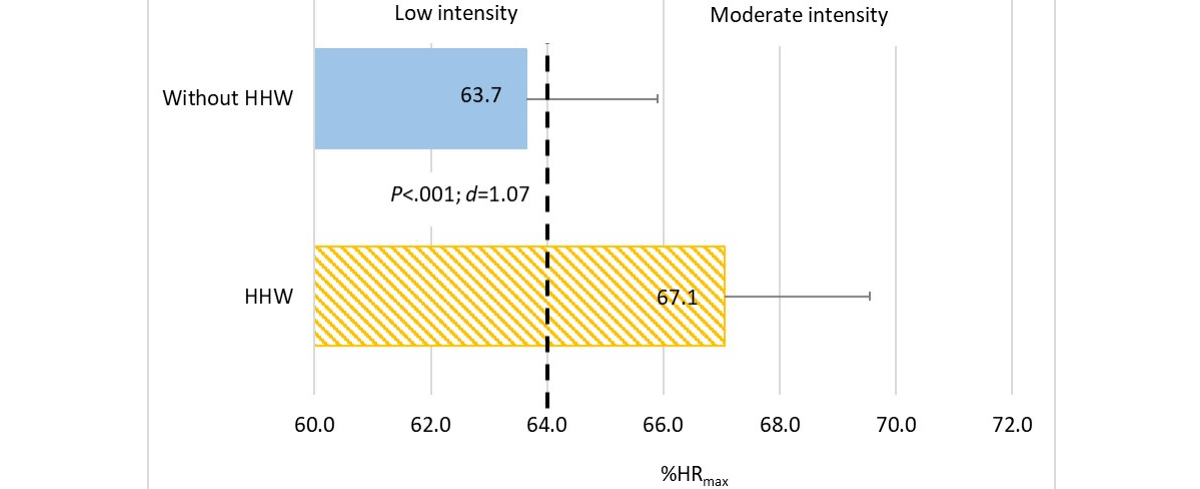
Intensity of physical exercise during playing Beat Saber depending on arm loading. % HR_max_: percentage of maximal heart rate; *d*: Cohen *d* value; HHW: handheld weight.

Heart rate measurements showed that the average heart rate (HR_avg_) of Beat Saber users playing without HHW was 123.2 (SD 18.2) bpm and was significantly lower (*P*<.001; Cohen *d*=1.7) than that observed when playing with HHWs (mean 129.8 bpm, SD 20.2 bpm). A similar statistically significant relationship was observed when analyzing the average percentage of maximum heart rate (% HR_max_). This parameter was also significantly lower (*P*<.001; Cohen *d*=1.07) when the arms were not loaded (mean 63.7% HR_max_, SD 9.3% HR_max_) than when participants played with HHWs attached to the wrists (mean 67.1% HR_max_, SD 10.3% HR_max_). It should be emphasized that in the conditions of no additional load, the intensity of PA was low, while with the upper limb load, it increased to a moderate level as recommended for health benefits (Figure 4).

**Figure 5:**
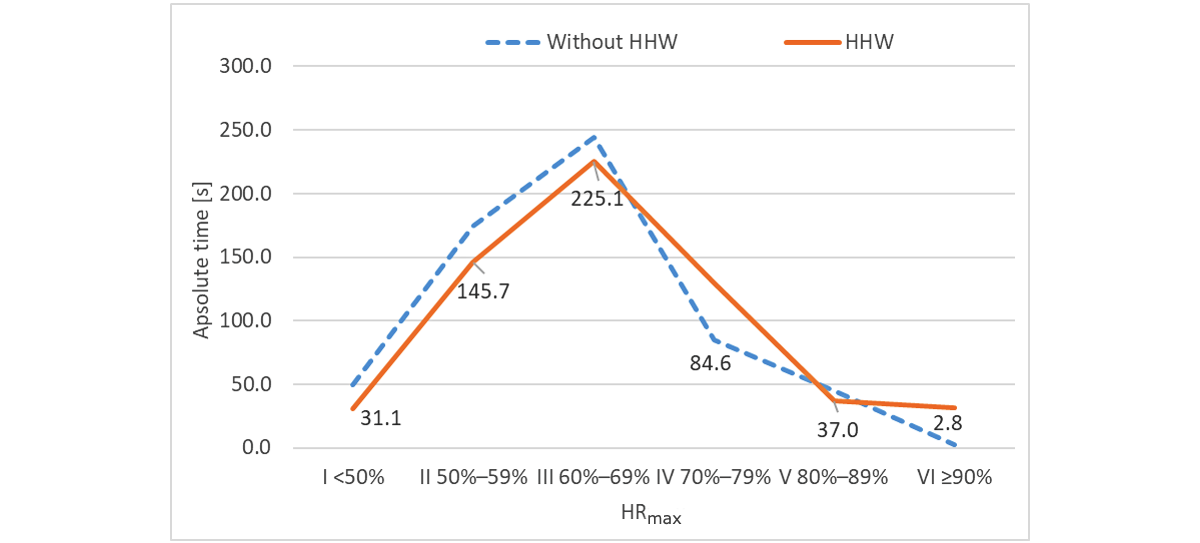
Average total time spent in different heart rate zones by participants during playing Beat Saber games with and without arm loading. HHW: handheld weight; HR_max_: maximal heart rate.

During both 10-minute sessions, participants' heart rates remained for the longest time within the range of 60%-69% HR_max_, which is optimal for fat reduction. When playing without HHW, the exercise at this intensity lasted 225.1 s whereas when playing with HHW on the wrists, users maintained this level of heart rate for 244.2 s. A comparison of the time spent in each heart rate zone in both sessions reveals that the greatest discrepancy was observed in zone IV (45 s). However, this difference was not statistically significant (Figure 5).

**Figure 6:**
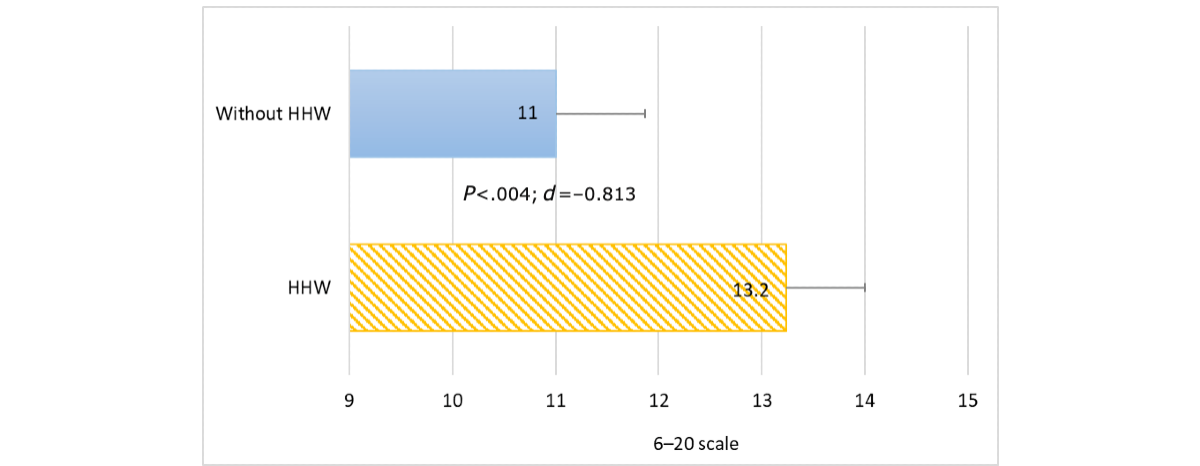
Rating of perceived exertion (6-20 scale) depending on arm loading. *d*: Cohen *d* value; HHW: handheld weight.

In the subjective perception of PA intensity made by study participants on the Borg RPE 6-20 scale, playing without HHWs was also reported to be significantly less intense (*P*=.004; Cohen *d*=–0.813) than playing with HHWs, with an average 11.0 (SD 3.2) points and 13.2 (SD 3.6) points, respectively (Figure 6). A comparison of these values with the classification of PA intensity [44] shows that the participants rated the intensity of exercise without HHWs as low and with HHWs as moderate, which was similar to the objective evaluation using a heart rate monitor. Furthermore, the analysis of correlations between subjective and objective measures of exercise intensity revealed a statistically significant (*P*<.001) high correlation (*r*=0.56) between intensity estimated based on % HR_max_ and the Borg RPE 6-20 scale for PA in VR with HHWs. In contrast, no such correlations were observed (*r*=0.08) when playing without HHWs.

### Attractiveness and Usability of an AVRG (Beat Saber) in the Opinion of Users and Comfort of Exercising With Loaded Arms

The results of the survey showed that the Beat Saber game was attractive and enjoyable for all study participants. This statement was agreed or strongly agreed upon by 100% (17/17) of the respondents. Nearly all (16/17, 94%) declared the willingness to be involved in regular PA using VR. Only 1 person was completely unwilling to do so. All respondents would or would rather recommend practicing PA in VR to others. Respondents were more divided in their opinions regarding the comparison of enjoyment of practicing PA in VR with conventional exercising. For the majority (10/17, 59%) of respondents, exercising in VR was more enjoyable than was conventional PA. The opposite view was held by 12% (2/17) of respondents. In contrast, 29% (5/17) of respondents did not express a clear opinion on this issue. Most respondents (14/17, 82%) believed that practicing PA in VR can complement a person's leisure-time PA. One respondent disagreed with this statement, and one had no opinion at all. To the question “Do you think practicing PA in VR can satisfy a person's needs for leisure-time PA?”, 76% (13/17) of respondents answered affirmatively. Furthermore, 12% (2/17) of respondents had no opinion on this issue, and 12% (2/17) disagreed with this statement. Almost half of the respondents (8/17, 47%) believed or tended to believe that practicing PA in VR could replace typical real-world forms of leisure-time PA, 12% (2/17) of the respondents were undecided on this issue, while the rest (7/17, 41%) had the opposite opinion. According to the survey, HHWs (0.5 kg) attached to the wrists did not disturb Beat Saber users. Only 1 player (1/18, 6%) had a rather different opinion (Table 1).

## Discussion

### Principal Findings

For several years, research has been conducted on the potential of active video games in engaging the musculoskeletal and cardiorespiratory systems as a health-promoting form of PA [16,22,45-47]. This is true for both nonimmersive and immersive VR applications, as AVRGs are a beneficial alternative to conventional video games, which are responsible for prolonged periods of sedentary behavior [48-51]. Furthermore, users from different age groups have indicated that such games are highly attractive and useful [15,20,25,52-54]. Unfortunately, many popular AVRGs are created to be used in a limited space, with controllers and HMDs primarily involving arm and torso movements while sitting or standing, which may translate into insufficient exercise intensity to provide health benefits. Although exercise intensity can be increased using trainers available on the market that are compatible with VR devices, their high prices and the required space limit their widespread use. Therefore, one should look for cheaper and more effective ways to increase PA intensity in VR.

To meet the aforementioned needs and expectations, it was hypothesized that a small additional load on the upper limbs of a 0.5-kg HHW and using a room-scale tracking mode would be a simple solution to increase the PA intensity of players. To our knowledge, no studies have been undertaken to date that have evaluated the effect of external arm loading on exercise intensity in young adults playing in VR. Bridging this gap in scientific research has been the direct objective of our analysis.

This study showed that the use of HHWs can be an effective solution. Relatively small and light weights (0.5 kg) contributed to a significant increase in PA intensity, expressed as an average % HR_max_ during playing Beat Saber, from low to moderate levels; that is, those recommended by the WHO for health benefits [31].

Although there is a lack of reports on exercising with HHWs in VR, our results can be related to studies of other forms of PA that have evaluated the effect of the external loading of the upper limbs on exercise intensity. Lee and Kim [55] demonstrated that the use of HHWs during exercise can increase the energy expenditure (EE) of upper body activity, which is extremely important for individuals with low fitness levels and special patient populations who cannot run, do not want to run, or have limited mobility of brisk walking. However, there are other studies in the available literature that do not support a significant effect of walking on a treadmill with HHWs on EE and other exercise parameters [37,38,56]. Furthermore, the use of heavier poles in Nordic walking was observed to have no effect on EE compared to Nordic walking with regular poles although greater muscle activity was reported [57]. However, it should be noted that Nordic walking has a higher EE compared to conventional walking [58]. This is probably largely due to the different ways of moving, but the weight of the poles held in the athlete’s hands is probably nonnegligible.

One important aspect of our study was that participants made the subjective assessment of PA intensity using the Borg RPE 6-20 scale. It was found that playing without HHWs was perceived as significantly less intense than playing with HHWs. A comparison of the obtained values to the classification of PA intensity [59,60] shows that the participants rated the intensity of the exercise without HHWs as low and with HHWs as moderate. Therefore, the subjective perceptions of PA intensity made by the participants in this study were consistent with the objective evaluation using a heart rate monitor. This could indicate that users engaged in PA in immersive VR can accurately assess their fatigue. However, this is not supported by the correlation analysis between subjective perceptions and objective measurements. It was demonstrated that a statistically significant relationship between intensity scores estimated based on % HR_max_ and the Borg RPE 6-20 scale was only present for practicing PA in VR with HHWs (*r*=0.56; *P*<.001). When playing without HHWs, no correlation (*r*=0.08) was found between % HR_max_ and the Borg RPE 6-20 scores. Similar findings were reported in a study that evaluated PA in VR in obese children during the playing of 2 AVRGs using an omnidirectional treadmill [22]. In the aforementioned studies, the games were played without any additional load, and the results of the subjective perceptions and objective measurements of PA intensity did not correlate with each other although the children in the study were able to accurately assess which game was more intense for them. This is difficult to interpret conclusively although it is hypothesized that the use of light additional resistance in VR makes the subjective perceptions of fatigue more relevant to the actual one than when PA is performed without a load. This can be surmised from studies showing that VR reduces the intensity of various stimuli (eg, pain) by distracting attention from thinking about the problem [61-64]. This is associated with what is known as a cognitive distraction. Perhaps a similar effect occurs during PA and helps alleviate the discomfort associated with intense exercise. This could be important for people with reduced physical fitness and poorer physical capacity, as they would be able to perform longer and more intense exercises in VR. This thesis is indirectly confirmed by the aforementioned studies of obese children who, despite declared low physical fitness, were able to exercise in VR for several minutes at a very high intensity (83% HR_max_) [22]. The usefulness of VR in distracting from unpleasant bodily sensations occurring during aerobic PA in overweight and obese children has also been demonstrated by other authors [65]. There are also reports indicating that VR reduces the negative sensations associated with performing isometric exercises, thus increasing the likelihood of performing them for longer periods of time [66]. Given the findings of these studies, there is much to suggest that VR may be useful in reducing the perception of exercise intensity and fatigue. However, providing unequivocal evidence for this phenomenon requires that further similar studies be undertaken.

The survey conducted in our study shows that the Beat Saber game was positively received by the respondents. All participants found it an attractive, enjoyable, and recommendable form of PA. Furthermore, almost all of them declared their willingness to regularly practice this form of PA in VR. The positive user reception of AVRGs has been also highlighted by other authors [16,22,52]. It is noteworthy that for the majority of respondents, PA in VR was more enjoyable than was conventional PA. Similar findings also emerged from another study that compared real-world and VR training on a cycle ergometer [18,20,25]. The participants reported having more fun riding a stationary bike in VR than in conventional conditions, which might have resulted from the gamification effect. Many studies have shown that satisfaction is an important predictor of engagement in PA regardless of participants’ age or health status [67-72]. In this context, AVRGs can be considered a form of exercise that can complement a person’s leisure-time PA and even satisfy their needs for physical exercise as indicated by the vast majority of respondents. Almost half of the respondents even claimed that PA in VR could replace typical real-world forms of leisure-time PA. It should be noted, however, that from the standpoint of inclusion, participation in social life, and the values of outdoor PA, it would be inadvisable to completely replace typical forms of PA in favor of those practiced in VR. Therefore, AVRGs should be considered complementary to conventional forms of PA.

An important aspect of the present study was also to assess any discomfort resulting from exercising with HHWs. According to the survey, additional HHWs (0.5 kg) attached to the wrists did not disturb Beat Saber players. Furthermore, given that it significantly increases PA intensity, HHW should be recommended for PA in VR based mainly on arm work. This can improve the effectiveness of the exercises and increase their health benefits. An important consideration here is the choice of the optimal load. This is because there is a concern that an excessive increase in exercise intensity during playing AVRGs may make playing less attractive. However, according to previous research, AVRGs that require more physical effort may be more appealing than may games characterized by lower-intensity exercise. For example, Dębska et al [16] examined user satisfaction with practicing PA in VR on the Omni omnidirectional treadmill and the Icaros flight simulator. Although exercise intensity was high in the former and low in the latter, the AVRG on the treadmill was rated higher in terms of attractiveness. Similar relationships have been observed in studies of obese children that compared the attractiveness of 2 AVRGs on a treadmill [22]. Again, higher PA intensity proved more attractive to users. This demonstrates that according to users, intense physical exercise in VR remains very attractive. To the best of our knowledge, which is based on our literature review, the research we conducted on the effect of HHWs on exercise intensity in VR can be considered novel. Although manufacturers of VR sets strive to minimize equipment and reduce its weight, it may be worth doing the opposite in some cases. Arm loading may be advisable not only to increase the intensity of exercise in VR, but also to enhance immersion (such as playing virtual tennis, baseball, or fighting with a heavy sword). Therefore, it seems warranted to undertake further multifaceted research on the effects of different sizes and different types of arm loading on the PA and the perceptions of players immersed in a virtual environment.

### Limitations

There is no doubt that the results presented in the study have several limitations. Given the relatively small group of participants, the results should be treated with caution. A more precise method of evaluating exercise intensity, such as indirect calorimetry, would be useful in future studies. In the present study, % HR_max_ was used because it was feared that the masks used in calorimetry might cause discomfort and lead to the participant's underestimation of the subjective appeal of the game, making it difficult to assess the comfort of performing exercises with HHWs. Furthermore, the use of our author-made survey questionnaire in the study is also a limitation, as it makes it difficult to accurately compare the findings regarding the enjoyment and attractiveness of using AVRGs with those published in previous studies. However, it should be noted that the questionnaire was only an additional component of this study.

### Conclusions

Our research, conducted on a group of young adults, showed that the AVRG Beat Saber is a form of PA that is attractive to users and can complement a person's leisure-time PA. It can also be a beneficial alternative to typical video games played in a seated position. With the additional loading of the participant’s wrists using a small HHW (0.5 kg), the relatively low intensity of physical exercise when playing Beat Saber (a game based primarily on arm movements) significantly increased to a moderate level, which in the context of health-related recommendations should translate into health benefits. Since PA in most modern AVRGs primarily involves upper limb movements, the use of HHWs seems to be a simple and effective way to increase exercise intensity, especially because, as reported by the study participants, such a procedure does not cause discomfort while using the application. Our findings may provide guidance to VR equipment manufacturers on how to make exercise more effective in the playing of AVRGs. However, to confirm the above conclusions, further research is needed using various existing applications that allow for the performance of PA in an immersive virtual environment. Future research would also benefit from evaluating the effect of changing the magnitude of arm loading on exercise intensity in VR. The use of a different type of arm load (eg, in the form of elastic resistance) seems equally interesting. In conclusion, we recommend the use of a light arm load in the form of HHWs when practicing Beat Saber AVRGs, as this increases the intensity of PA, which may translate into health benefits.

## Abbreviations

% HR_max_: percentage of maximal heart rate
AVRG: active virtual reality game
EE: energy expenditure
HHW: handheld weight
HMD: head-mounted display
HRavg: average heart rate
HR_max_: maximum heart rate
PA: physical activity
RPE: Rating of Perceived Exertion
VR: virtual reality
WHO: World Health Organization
